# Examining Variations in Surfactant Administration (ENVISION): A Neonatology Insights Pilot Project

**DOI:** 10.3390/children8040261

**Published:** 2021-03-28

**Authors:** Priya Patel, Andrew Houck, Daniel Fuentes

**Affiliations:** Chiesi USA Inc., 175 Regency Woods Place, Ste. 600, Cary, NC 27518, USA; priya.patel@chiesi.com (P.P.); Andrew.Houck@chiesi.com (A.H.)

**Keywords:** surfactant, respiratory distress syndrome, neonatology, clinical practice variation, INSURE, intubation, premedication, guideline, non-invasive respiratory support, protocol

## Abstract

Variability in neonatal clinical practice is well recognized. Respiratory management involves interdisciplinary care and often is protocol driven. The most recent published guidelines for management of respiratory distress syndrome and surfactant administration were published in 2014 and may not reflect current clinical practice in the United States. The goal of this project was to better understand variability in surfactant administration through conduct of health care provider (HCP) interviews. Questions focused on known practice variations included: use of premedication, decisions to treat, technique of surfactant administration and use of guidelines. Data were analyzed for trends and results were communicated with participants. A total of 54 HCPs participated from June to September 2020. In almost all settings, neonatologists or nurse practitioners intubated the infant and respiratory therapists administered surfactant. The INSURE (INtubation-SURrfactant-Extubation) technique was practiced by 83% of participants. Premedication prior to intubation was used by 76% of HCPs. An FiO_2_ ≥ 30% was the most common threshold for surfactant administration (48%). In conclusion, clinical practice variations exist in respiratory management and surfactant administration and do not seem to be specific to NICU level or institution type. It is unknown what effects the variability in clinical practice might have on clinical outcomes.

## 1. Introduction

Respiratory distress syndrome (RDS) is one of the most common diagnoses in premature infants, primarily resulting from surfactant deficiency and lung immaturity. Advances in clinical care including the use of antenatal corticosteroids, surfactant and early continuous positive airway pressure (CPAP) have had a substantial impact on reducing morbidity and mortality [1,2]. Increased adherence to care practices have been associated with improved neonatal outcomes [3]. However, while practice guidelines exist, there is a lack of high-quality evidence to consistently guide many practices used in neonatal respiratory management [1]. In the most recent American Academy of Pediatrics (AAP) Clinical Report pertaining to surfactant administration, the authors conclude that due to conflicting and limited data, the optimal method of surfactant administration in preterm infants has yet to be clearly identified and there is insufficient evidence to recommend an optimal number of fractional doses of surfactant or what body position is best when surfactant is administered [2]. Similarly, guidelines developed specifically for registered respiratory therapists (RRTs) also do not address specific details for surfactant administration [4]. As a result, HCPs in neonatal intensive care units (NICU) often create and adhere to their own protocols based on experience, product information and their own interpretation of clinical literature. Recent studies have examined a wide variation in respiratory management in preterm infants within and across international and national networks. The impact of this variability in practice is unknown [1,5]. The goal of this survey-based pilot project was to better understand and catalog clinical practice variability in the management of RDS in the US.

## 2. Materials and Methods

A 30-question survey was developed to describe and understand known clinical variations in neonatal respiratory management and surfactant administration (Appendix A). Topics were derived from various sources including HCP discussions, medical information requests, recurring medical conference lectures and recent publications. Along with determining what clinical practice variations exist, an emphasis was placed on understanding why these variations occur. Questions pertaining to surfactant administration were not specific to any surfactant drug product and identification of surfactant(s) used in the respective institutions was not requested as a part of this survey. The target survey participant was defined as any HCP directly involved in procedures to intubate and administer surfactant in the NICU; this primarily included neonatologists, neonatal fellows, neonatal nurse practitioners (NNPs) and RRTs.

The survey was developed by the Medical Affairs department at Chiesi USA, Inc. (Cary, NC, USA) The Chiesi USA Special Care Medical Affairs Department includes four field-based Medical Science Liaisons (MSLs) that support the neonatal therapeutic area for the organization. The Chiesi MSL team is comprised of three pharmacists (Pharm.D.) and an RRT, all with extensive clinical practice backgrounds in respiratory care and/or neonatology. The MSLs primary role is to communicate scientific and clinical information to the medical community in a strictly non-promotional capacity. Health care practitioners in the field of neonatology who had either previously submitted information requests to the Medical Affairs Department or with whom an existing professional relationship was present were identified by a non-random convenience sampling method. Team members then proactively contacted the HCPs to ascertain their willingness to participate in the survey. No compensation was provided to participants. The survey was conducted as individual face to face meetings utilizing video conferencing (or on rare occasion a phone call) in an interview format utilizing Microsoft Forms (Microsoft Office 365 E3, Redmond, WA) to present questions and record data. Meeting times averaged approximately 45 min for survey completion. Response options were provided mostly as multiple choice or as “check all that apply”, with an option of “other” for free text response. Participants were also provided with an option to decline responding to individual questions at their discretion. All data collected was kept anonymous and for the purposes of confidentiality, participant identities and hospital names were not collected or linked to the answers. Prior to commencing the survey, participants were to acknowledge and agree that responses would be stored in a deidentified manner and would not be linked to the individual participant. Descriptive statistics were used to evaluate responses and results were shared with individual participants for informational purposes only. Because there was never an intent to identify specific individual respondents or specific patients, a decision was made that a priori submission of the project to review by an ethical research committee was not required.

## 3. Results

From June to September 2020, a total of 75 HCPs were invited to participate in the ENVISION survey, of which 54 agreed to participate. Participating HCPs included 33 neonatologists, 17 RRTs, three NNPs and one neonatal fellow. Many HCP participants practice at several NICUs, ranging from 15 to 142 beds. HCPs reported working exclusively or a majority of their time at academic centers (*n* = 33), followed by community-based hospital (*n* = 20), with one identifying as equally divided. Fifty individual institutions in 26 US states were represented, four of which had more than one participant in the survey. Level of NICUs were reported as the highest level of care where HCPs practiced. This represented 24 level IV, 25 level III and one level II NICUs (Table 1).

### 3.1. Initial Respiratory Management

The results show nine different combinations were reported when participants were asked which methods of initial respiratory support management were utilized in their clinical practice. Among the 54 HCPs, 54% (*n* = 29) identified a single method, 41% (*n* = 22) identified a combination of two methods as their initial management practice, with 5% (*n* = 3) identifying a combination of three methods as initial management. CPAP use was reported by 96% (*n* = 52) of the participants. The type of CPAP varied and the following percentages were reported as options for use (either alone or as an alternative to another method): 42% bubble CPAP (*n* = 22/52), 44% nasal CPAP (*n* = 23/52) and 13% utilized both (*n* = 7/52). Other methods that were noted for initial management were 33% (*n* = 18) non-invasive positive pressure ventilation (NIPPV), 6% (*n* = 3) high flow nasal cannula (HFNC) and 4% (*n* = 2) neurally adjusted ventilatory assist (NAVA). It was common for participants to note multiple methods of respiratory management making it difficult to evaluate trends across institution type and NICU level.

The most common treatment of infants ≤ 26 weeks gestational age (GA) was early non-invasive respiratory support and surfactant administration reported by 63% (*n* = 34) of HCPs. However, 24% (*n* = 13) place these infants on MV after surfactant administration. An FiO_2_ threshold of ≥ 30% for surfactant administration was identified by 48% (*n* = 26) of HCPs that participated. Academic hospitals (55%, *n* = 18/33) and level IV NICU’s (54%, *n* = 14/26) more commonly used an FiO_2_ threshold of ≥ 30% for surfactant administration as compared to community hospitals (40%, *n* = 8/20) and level III NICU’s (44%, *n* = 12/27). That other criteria in addition to FiO_2_ needed to be considered was reported by 24% (*n* = 13) of participants. Considerations that were noted by HCPs were GA, PaO_2_, range of FiO_2_, type of respiratory support and/or type of surfactant administration.

### 3.2. Administration/Intubation Practices

The most-frequently reported provider-type involved in the intubation of neonates were neonatologists and NNPs, 94% (*n* = 51) and 93% (*n* = 50), respectively, followed by RRTs at 56% (*n* = 30). After intubation, the respiratory therapist was the most often involved in administration of surfactant 83% (*n* = 45), followed by the neonatologist 43% (*n* = 23). These results were similar when comparing NICU level as well as hospital type.

There was wider variability seen for other aspects of surfactant administration. A multi-access catheter was identified as the catheter used most often for administration at 60% (*n* = 32) (Table 2). When calculating the surfactant dose, 56% (*n* = 30) of HCPs administer the dose as calculated with 26% (*n* = 14) rounding to the nearest full vial size within a 10% limit. There was variability in those that rounded the dose with a mixture of providers that opt not to round down, while others opt not to round up. When asked about administration via a single bolus dose versus multiple aliquots, 50% (*n* = 27) use two aliquots, 32% (*n* = 17) use a single bolus dose, 7% (*n* = 4) use > 2 aliquots (*n* = 4) and 11% (*n* = 6) report other. The reasoning most often provided for using the single bolus dose was tolerability as well as maintaining a neutral position. For HCPs that practice using two aliquots, historical practice was most frequently noted with tolerability being second highest. Those HCPs that reported the “other” considered type of surfactant used, location, GA/weight and clinical presentation to determine how surfactant is administered. During administration, 44% (*n* = 24) of HCPs turn or reposition the infant. Most common reasons for turning the infant were surfactant distribution 67% (*n* = 36) and historical practice 46% (*n* = 25). Positive pressure ventilation (PPV) is used by 94% (*n* = 51) of HCPS when administering surfactant. A video laryngoscope was available to 80% (*n* = 43) of the participants; 2% (*n* = 1) reported using it always, 48% (*n* = 26) use only for difficult intubations or training and 30% (*n* = 16) do not use.

The practice of early administration of surfactant followed by brief ventilation and rapid extubation to noninvasive ventilation, otherwise known as INSURE was noted by 83% (*n* = 45) of HCPs. INSURE was utilized at 79% of academic hospitals (*n* = 26/33) and 90% of community hospitals (*n* = 18/20) surveyed. This use was similar between level III (85%, *n* = 23/27) and level IV (81%, *n* = 21/26) NICUs. Among those 45 HCPs that responded with “yes”, 62% (*n* = 28) extubate in 10 min or less (Table 3). Some HCPs noted a practice of extubation in less than 5 min after the dose. Challenges to using INSURE were reported by 61% (*n* = 33) of HCPs with factors such as no clear definition when extubation should occur, time for physiologic respiratory adaption, premedication effects and concern for harm.

**Figure 1:**
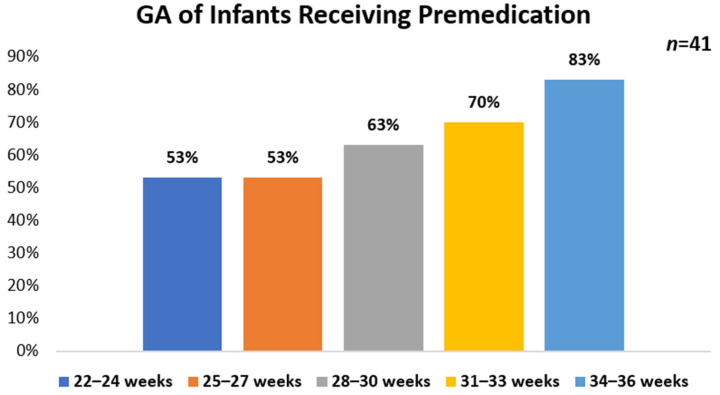
Gestational age of infants receiving premedication for intubation

The frequency of premedication use prior to intubation for surfactant administration was assessed in this survey. Overall, 76% (*n* = 41) of HCPs reported premedication use. Of those using premedication, 54% (*n* = 22/41) reported using a protocol while 46% (*n* = 19/41) do not. HCPs who primarily practice at academic hospitals represented the majority of participants who reported having a protocol 77% in place (*n* = 17/22) as compared with community-based hospitals 18% (*n* = 4/22). No protocol use (*n* = 19) was reported for a similar number of academic hospitals 58% (*n* = 11/19) and community-based hospitals 42% (*n* = 8/19). For participants reporting the use of premedication (*n* = 41), the highest use was found in infants who were 34–36 weeks GA (83%, *n* = 34), followed by 31–33 weeks GA (70%, *n* = 29), 28–30 weeks (63%, *n* = 26) and 22–27 weeks GA (53%, *n* = 22) (Figure 1). There were 48% (*n* = 20) of HCPs that use premedication in all infants. A majority of HCPs reported the NICU as the primary location that premedication is given 93% (*n* = 38).

Wide variability was seen in which agents were used, either singly or in combination. Overall, fentanyl was the most common premedication used at 73% (*n* = 30). Midazolam was the most common sedative used at 29% (*n* = 12). The use of neuromuscular blockers in combination with any analgesic and/or sedative agent was less common at 27% (*n* = 11). The most common reason noted for use of premedication was to prevent discomfort/pain in the infant, 76% (*n* = 31), followed by ease of laryngoscopy/intubation, 56% (*n* = 23).

### 3.3. Guideline and Protocol Use

Participants reported that hospital-based guidelines were most used 55% (*n* = 30) to guide clinical practice for RDS management. Guidelines published by the AAP influenced clinical practice in 44% (*n* = 24) of participants. Other combinations of guidelines/protocols that were described were varying mixtures of AAP, databases such as Vermont Oxford Network (VON) or Mednax, European consensus guidelines for surfactant administration and the American Association of Respiratory Care (AARC) [2,4,6].

The most recent version of the AAP guidelines on surfactant replacement therapy for preterm and term neonates with RDS was published in January of 2014 [2]. We asked HCPs their thoughts on whether or not these guidelines should be updated. Of the HCPs surveyed, 65% (*n* = 35) of participants strongly agreed that the guidelines should be updated. Additionally, HCPs commented on specific areas which should be addressed in future guidelines, shown in Table 4.

## 4. Discussion

This pilot survey examining RDS management and surfactant administration practices in US NICUs uncovered wide practice variations as well as some similarities. Data from multiple randomized control trials (RCTs) and meta-analyses demonstrate that the routine use of CPAP significantly reduces mechanical ventilation and the outcome of bronchopulmonary dysplasia (BPD) or death in at-risk preterm infants [7]. However, multiple variations exist with CPAP utilization including the technology used to provide positive-pressure (ventilator or bubble CPAP), pressure settings, as well as the type of interfaces (prongs or mask) [8]. These variables are often difficult to tease out in clinical studies and have not been directly compared in large RCTs. Nasal CPAP as an initial respiratory method was used by 55% of participating HCPs. Alternative methods of respiratory support such as HFNC, NIPPV and NAVA have also been evaluated in clinical trials, but research in a larger group of infants is still necessary to compare different modalities and identify sub-populations that may potentially have the most benefit. Our results confirm that many NICUs have multiple methods of respiratory support available, 46% report >1 method for initial respiratory management. A recent meta-analysis concluded that NIPPV appears to be the most effective noninvasive respiratory support method in preterm neonates with RDS to prevent mechanical ventilation and respiratory failure in the first few days of life [9]. However, authors note the quality of evidence in all of the included studies were very low to moderate for the primary outcomes. In our study, NIPPV use was reported as an initial method of support by 33% of participating HCPs. Although avoidance of invasive mechanical ventilation is a common goal to reduce lung injury, 24% of HCPs report its use in < 26 weeks GA and this number is likely higher when stratifying infants < 24 weeks GA.

The AAP recommends CPAP use immediately after birth with subsequent selective surfactant administration as an alternative to routine intubation with prophylactic or early surfactant administration in preterm infants [2]. Early surfactant (<2 h of age) in infants with RDS decreases the risk of mortality, air-leak and chronic lung disease [10]. This recommendation resulted from a meta-analysis that included six RCTs published from 1992–2003, two trials utilizing synthetic surfactant (Exosurf^®^) and four utilizing animal-derived surfactant preparations (Surfactant TA^®^, Aleveofact^®^ and Curosurf^®^) [10]. No specific clinical criteria for surfactant administration are addressed in the published US guidelines [2,4]. In contrast, the European Consensus Guidelines suggests a policy of early rescue surfactant and a protocol to treat infants who are worsening when FiO_2_ > 0.30 on CPAP pressure of at least 6 cm H_2_0 [6]. A threshold of FiO_2_ ≥ 0.30 for surfactant administration was reported by 48% of HCPs. However, almost a quarter of participants reported other criteria that are considered for surfactant administration including GA.

NEAR4NEOs is an international registry study conducted in academic centers [11]. First, providers for combined intubations in the NICU and delivery room (DR) were identified as advance practice providers (37%), neonatal fellows (35%), pediatric residents (12%), attending neonatologist (6%), RRTs (2%) and others (8%). Conversely, our data reflected neonatologists and NNPs to be the main providers for intubation with RRTs providing surfactant administration. Initially, we hypothesized discrepancies may be due to the NEAR4NEOs data reflective of practices in academic only centers and differences in international providers. However, further analysis of NICU type and level did not reveal any differences. Of note, our data are representative of only NICUs in the United States. The AAP defines levels of neonatal care as Level I (well newborn nursery), Level II (special care nursery), Level III (NICU) and Level IV (Regional NICU) [12]. The majority of our survey participants report working at level III and/or level IV NICUs. Level III units provide care to infants <32 weeks GA and <1500 g with a full range of respiratory support that may include conventional and/or high-frequency ventilation and inhaled nitric oxide. Level IV units have the additional capabilities and extensive experience in the care of the most complex and critically ill infants with pediatric medical and surgical specialists continuously available. Unit-level variations in HCP availability has been previously reported by the International Network for Evaluating Outcomes (iNeo) [13]. The presence of a continuously available respiratory therapist in the NICU is unique to the US and Canada and may not be the practice in other countries. A multi-access catheter was the most frequently used catheter 60% (*n* = 33) for surfactant administration. We recognize that further questioning would have been beneficial to ensure that there was no overlap due to discrepancies in terminology with functionally similar catheters. For this reason, we chose to report catheters exactly how participants reported without interpretation.

Surfactant specific product information often provides details for administration such as type of catheter, single bolus dosing vs multiple aliquots and recommendations for body positioning [14,15,16]. Supporting clinical evidence is limited and often decisions are made at HCP discretion based off historical practice, perceived surfactant distribution and tolerability. Video laryngoscopy provides the HCP with a shared airway view when intubating, but results are inconsistent for rates of intubation success during training for neonatal trainees and inexperienced HCPs [17,18,19]. Insufficient clinical evidence exists to compare video laryngoscopy to direct laryngoscopy for intubation success [20]. However, video laryngoscope use has been associated with reduced odds of tracheal-intubation associated events [11]. Although 80% of participants had access to a video laryngoscope, many only use it for difficult intubations or training (48%) and some do not use at all (30%).

The INSURE strategy is being utilized worldwide on the basis of increased supportive evidence [2,21]. Our findings show that 83% of HCPs utilize the INSURE technique, however, we did not determine the regularity with which it was used or identify a specific sub-population of infants in which it is utilized. In clinical studies of INSURE, infants were generally extubated within 5 to 10 min [22,23,24]. Similarly, 62% of participants report extubating within 10 min and some reported extubation in less than 5 min. INSURE failure rates of 9–50% have been reported in clinical studies dependent on the definition of failure and study population suggesting that it may be beneficial in certain sub-populations [25,26,27]. ELBW, low GA and severe RDS have been identified as risk factors for INSURE failure [28,29,30]. While 39% of HCPs report no challenges associated with INSURE, 27% reported a concern for harm. We were unable to determine the exact concerns from harm during our data collection, although previous studies have referred to INSURE failure, specifically the consequences of delayed mechanical ventilation [31].

AAP Recommendations suggest premedication be used for all non-emergent intubations to eliminate pain/discomfort, injury to the airway and physiologic instability [32]. Empirical evidence also shows an increase in intubation success, time to intubate and decreased complications associated with the procedure. However, this still remains controversial amongst some HCPs and has been a slow transition into clinical practice as optimal agents, doses, combinations, long-term benefits and adverse effects remain unknown. Premedication use was reported by 76% (*n* = 41) of participants, similar to 52% premedication use seen combined in NICU and the delivery room (DR) in the NEAR4NEOs registry [11]. Only three HCPs (7%) reported use of premedication in the DR, while 14% were reported in NEAR4NEOs registry. Premedication use increases as GA increases with only 48% reporting use in all GA. Data for which agents were used as premedication resulted in many drug options within a drug class and variable combinations, resulting in a complicated analysis. Use of reversal agents to extubate after surfactant administration was used by any participating HCP.

As mentioned previously, several US guidelines exist related to respiratory support management and surfactant administration in the premature infant [2,4,33]. We hoped to determine what impact these guidelines and network databases such as the Vermont Oxford Network or Mednax have on clinical practice for RDS. A variety of resource combinations were reported with 55% (*n* = 30) of HCPs having their own hospital-based guidelines. AAP guidelines influenced clinical practice in 44% (*n* = 24) and 11% (*n* = 6) reported that no formal guideline was in place due to provider specific practice. Given that these guidelines have not been updated in 7–8 years, 65% of HCPs strongly agree (*n* = 35) and 30% agree (*n* = 16) that the RDS guidelines should be updated. Specific areas that should be addressed are listed in Table 2. Participants noted a variety of reasons why these guidelines are not regularly updated including lack of funding/support, need for US specific studies and the allowance for clinical judgement. In addition, it was noted how niche the neonatal population is, which makes it difficult to keep up with smaller studies and published literature. Conversely, those that feel the guidelines needed updating suggested surfactant replacement therapy had been mastered when initially launched in the late 90 s and surfactant is deemed safe; therefore, there is no driving need to look further.

There are several limitations to interpretation of the results of this project. Although the scope of the survey was intended to reflect respiratory distress syndrome management, some responses may have reflected surfactant use for other less common indications. Our data represent a small sample size of 54 participating HCPs and limited representation from neonatology fellows and NNPs. Since invitations for participation were extended first to those HCPs where professional relationships already existed, this created a potential source of bias in the selected sample. However, it should be noted that several HCPs participated in the survey as a follow-up to an independent submission requesting medical information. The diversity of our sample population may not have been in alignment with current stratification of NICU acuity and providers in the US which could have affected our results. Responder bias is common with survey studies and cannot be ruled out as answers may not have truly represented clinical practice. HCPs often work at more than one NICU which also could have biased answers to reflect intersystem variability. Answers reflected how HCPs provided clinical care the majority of time. We also recognize that allowing for an “other” free text option may have contributed to the variability in answers. However, in this unique population and as seen by our results there were many answers that we could not have anticipated. During data analysis, we limited interpretation of answers as much as possible. A few questions, however, did allow for additional analysis based on detailed HCP feedback. Although four NICUs had two different interdisciplinary HCPs participate, we did not compare responses within an institution. These findings would have been of additional interest when evaluating clinical practice variability. We acknowledge that a number of factors, including gestational age and clinical presentation, impact decisions made to administer certain elements of clinical care. The survey was intended to serve as an informative and hypothesis-generating tool and not to guide clinical decisions. Other relevant data points would help to further characterize variability of care. These may have included variables for RDS diagnosis, utilization of chest x-rays, details of non-invasive respiratory support (i.e., order of methods used, CPAP pressure settings, flow rates and types of interfaces used) and extubation readiness criteria. In addition, criteria used in the decision to treat with surfactant, time to surfactant administration and details for subsequent surfactant therapy would have been valuable. Respiratory management guidelines and/or surfactant protocols were not collected for analysis and may have provided further insight into practice variations. Our survey did not proactively solicit information on the use of emerging novel methods for surfactant administration, such as the use of less invasive surfactant administration (LISA) technique, aerosolization or a laryngeal mask airway. Although some of these methods are reported to be in common use outside the U.S., they have not been approved by the U.S. Food and Drug Administration (FDA) for any of the available surfactant drug products at this time. This may limit the interpretation of our findings. We acknowledge the importance of better understanding the use of these novel methods and will incorporate assessments into a future project.

The ENVISION project was deployed as a pilot study to characterize and provide insight on clinical practice variability in infants with RDS. We plan to utilize the results of this experience to conduct additional surveys in future projects.

## 5. Conclusions

We achieved our goal to describe variability amongst NICUs and provide a snapshot of current clinical practice for RDS management. Uniquely, this survey also characterized specific variability during surfactant administration which has not been previously reported. However, a larger sample of participants may provide more insights in identifying trends in these variabilities. In the absence of evidence, variability in clinical practice is common. The majority of HCP participants strongly agreed that updated recommendations for RDS management and surfactant administration are warranted.

## Figures and Tables

**Table 1:** Demographics.

**Demographics**	***N*= 54** *n* (%)
HCP type	
- Neonatologist - Respiratory Therapist - Neonatal Nurse Practitioner - Neonatology Fellow	33 (61%) 17 (31%) 3 (6%) 1 (2%)
Practice site	
- Academic Hospital - Exclusively Academic - Majority of time Academic - Community Hospital - Exclusively Community - Majority of time Community - Both (equal time)	33 (61%) 25 (46%) 8 (15%) 20 (37%) 11 (20%) 9 (17%) 1 (2%)
**Represented NICUs**	***N*= 50** *n* (%)
NICU acuity level	
- Level II - Level III - Level IV	1 (2%) 25 (50%) 24 (48%)
Bed size of NICU	
- <20 - 21–30 - 31–50 - 51–100 - >100	2 (4%) 7 (14%) 15 (30%) 22 (44%) 4 (8%)

HCP, health care provider; NICU, neonatal intensive care unit.

**Table 2:** Type of catheter used for surfactant administration.

Catheter Used	*n* = 55 *
Multi-access catheter	33
5-French catheter	9
Suction catheter	5
Side port of endotracheal tube (ETT)	3
5-French umbilical artery catheter	2
Feeding tube	2
16-gauge angiocath	1

* One HCP stated the practice of using a different type of catheter depending on if patient was in delivery room (DR) vs. NICU.

**Table 3:** Time to extubation after surfactant administration.

Time of Extubation after Surfactant	*n* = 45
<10 min	28 (62%)
10–30 min	14 (31%)
30–60 min	2 (5%)
>60 min	1 (2%) *

* > 60 min to extubation reported in a referral hospital.

**Table 4:** Specific areas suggested for inclusion in next update of AAP Guidelines.

Timing of surfactant therapy
Criteria for surfactant use
Surfactant use in infants of varying GA (e.g., larger infants, ELBW)
Early surfactant administration
Incorporation of new clinical evidence

ELBW, extremely low birth weight; GA, gestational age; AAP, American Academy of Pediatrics

## Data Availability

The data presented in this study are available on request from the corresponding author.

## Appendix A. Project ENVISION Survey Instrument

Instructions: Complete list of survey questions (all questions had a “prefer not to answer” option and the majority had an “other” option to capture free text)

1. Healthcare provider acknowledges and agrees that responses are stored only in a deidentified manner. Because the data being collected is deidentified, it cannot be linked back to the participant.
  - I acknowledge and agree to providing my responses.
  - I do not wish to participate
2. Participant Characteristics (multiple choice)
  - Neonatologist
  - Neonatal Fellow
  - Respiratory Therapist
  - Neonatal Nurse Practitioner
3. Hospital Practice Site (multiple choice)
  - Academic Medical Center
  - Community Hospital
  - Both (majority in academic)
  - Both (majority in community)
  - Both (evenly divided)
4. Location of Hospital (City, State)
5. Level of NICU (Multiple choice)
  - Level II
  - Level III
  - Level IV
6. Approximate NICU Bed Size (fill in the blank)
7. Who intubates and administers surfactant? (Check all that apply)
  - Neonatologist
  - Neonatal fellow
  - Respiratory Therapist
  - Neonatal Nurse Practitioner
8. Do you have a standardized protocol for the use of premedication(s) for intubations for surfactant administration? (multiple choice) [If not using premedication, skip to question 14]
  - Yes
  - No
  - We do not use premedication
9. When intubating for the purpose of surfactant administration, what premedication(s) are administered? (check all that apply)
  - Atropine
  - Fentanyl
  - Morphine
  - Remifentanil (Ultiva)
  - Vecuronium (Norcuron)
  - Rocuronium (Zemuron)
  - Pancuronium (Pavulon)
  - Cisatracurium (Nimbex)
  - Midazolam (Versed)
  - Propofol (Diprivan)
  - Dexmedetomidine (Precedex)
  - Succinylcholine
  - Sucrose
10. Where are premedication(s) used? (multiple choice)
  - Delivery Room
  - NICU
  - Both
11. Which babies receive premedication(s) prior to intubation for surfactant administration and subsequent ventilation? (Check all that apply)
  - 22–24 weeks
  - 25–27 weeks
  - 28–30 weeks
  - 31–33 weeks
  - 34–36 weeks
  - >36 weeks
  - All babies
12. Do you routinely utilize any reversal agents to extubate patients faster after surfactant administration? (multiple choice)
  - Yes
  - No
13. What drives your decision to give premedication(s)? (check all that apply)
  - Ease of laryngoscopy/intubations
  - Discomfort/Pain of neonate
  - Peer reviewed clinical guidelines (AAP recommendations) /EBM
  - Historical practice
14. If you do not use premedication(s), why? (check all that apply) [Only for those that do not give premedication]
  - Respiratory depression- delays extubation
  - Not enough evidence for long-term effects of premedication
  - Access to medications
  - Historical Practice
15. Do you use a video laryngoscope for intubation? (multiple choice)
  - Yes—always
  - Yes—sometimes only for difficult intubations or training
  - No
  - No- we do not have access to this technology
16. When calculating the weight-based surfactant dose for administration, what is the most common practice? (multiple choice)
  - Administer dose as calculated
  - Administer dose rounded to the nearest full vial size
  - Administer dose rounded to the nearest full vial size (within a 10% limit)
  - Administer dose per dosing card
  - Surfactant prepared by pharmacy
  - Unknown
17. What type of catheter is most commonly used to administer surfactant? (multiple choice)
  - 5 Fr catheter
  - Multi-access catheter (MAC)
  - No catheter straight down the ETT
18. Do you use Positive Pressure Ventilation (ventilator, Neopuff, bag/mask etc) to administer the surfactant? (multiple choice)
  - Yes
  - No
19. Do you mainly use a single bolus dose or multiple aliquots? (multiple choice)
  - Single bolus
  - Two aliquots
  - More than two aliquots
20. What is the decision to give a single bolus vs multiple aliquots based on? (check all that apply)
  - Maintaining a neutral position
  - Surfactant volume and viscosity
  - Surfactant labeling
  - Better tolerated
  - Less reflux of surfactant
  - Historical Practice
21. Do you turn the baby during surfactant administration? (multiple choice) [If no, skip to question 23]
  - Yes
  - No
22. If yes, what is the decision to turn based on? (check all that apply)
  - Surfactant distribution
  - Surfactant labeling
  - Better tolerated
  - Historical Practice
23. Do you practice the INSURE method? (multiple choice) [If no, skip to question 25]
  - Yes
  - No
24. If yes, on average how rapidly is the baby extubated? (multiple choice)
  - <10 min
  - 10–30 min
  - 30–60 min
  - >60 min
25. If any, what are the hurdles to using the INSURE method? (check all that apply)
  - None
  - There is no clear definition of when extubation should occur with INSURE
  - Does not allow for enough time for physiologic respiratory adaption
  - Concern for harm from extubating too early
  - Lack of resources to support rapid extubation
  - Not familiar with this technique
  - Not enough clinical evidence in the US to support use
  - Duration and effect of premedication
26. What method(s) of initial respiratory management correlates with your clinical practice? (check all that apply)
  - nCPAP (nasal CPAP)
  - Bubble CPAP
  - HFNC (high flow nasal cannula)
  - NIPPV (non-invasive positive pressure ventilation)
  - MV (mechanical ventilation)
27. Along with other clinical factors, at what FiO_2_ threshold is surfactant administered? (check all that apply)
  - FiO_2_ ≥ 30%
  - FiO_2_ ≥ 40%
  - FiO_2_ ≥ 50%
  - FiO_2_ ≥ 60%
28. What is initial respiratory management for a preterm infant ≤ 26 wk GA? (multiple choice)
  - Early NIV and selective surfactant
  - Very early rescue surfactant via INSURE in the DR and then place on NIV
  - Very early rescue surfactant admin in the DR and then place on mechanical ventilation
  - Early rescue surfactant via INSURE in the NICU and then place on NIV
  - Early rescue surfactant admin in the NICU and then place on mechanical ventilation
29. What guidelines do you currently follow in clinical practice for RDS? (check all that apply)
  - American Academy of Pediatrics (AAP) guidelines
  - American Association of Respiratory Care (AARC) guidelines
  - Hospital-based guidelines
  - Database driven (i.e., VON, Mednax)
30. Given the published US surfactant replacement therapy guidelines were last updated 6–7 years ago. Is there a need for these type of guidance documents to be updated on a regular basis reflecting current literature and standard of care? (multiple choice)
  - Strongly Agree
  - Agree
  - Neutral
  - Disagree
  - Strongly Disagree
